# Influence of negative emotions on residents’ learning of scientific information: an experimental study

**DOI:** 10.1007/s40037-019-00525-8

**Published:** 2019-07-23

**Authors:** Telma Kremer, Silvia Mamede, Walter W. van den Broek, Henk G. Schmidt, Maria do P. T. Nunes, Milton A. Martins

**Affiliations:** 1 Institute of Medical Education Research Rotterdam, Erasmus Medical Centre, Rotterdam, The Netherlands; 2 Department of Internal Medicine, School of Medicine, University of São Paulo, São Paulo, Brazil

**Keywords:** Emotions, Learning, Medical education, Cognition

## Abstract

**Introduction:**

Medical training is consistently described as emotionally challenging. Students commonly encounter situations that are likely to trigger emotional reactions, but the influence of emotional reactions to these situations on learning is unclear. This experiment examined the effects of negative emotions on medical residents’ learning of scientific information.

**Methods:**

Sixty first-year internal medicine residents (i.e. physicians in training to become specialists) at the São Paulo University Medical School were randomly assigned to watching a video clip either presenting an emotional (experimental group) or a neutral (control group) version of the same situation. Subsequently, all residents studied the same scientific text. Main outcome measurements were learning processes (inferred through study time and cognitive engagement) and outcomes (recall accuracy). Data were analyzed using chi-square and independent t‑tests.

**Results:**

The experimental group spent significantly less time (*p* < 0.001) studying the text and performed significantly worse on the free recall test (*p* < 0.001) than the control group.

**Discussion:**

Negative emotions decreased time invested in a learning task and the amount of knowledge gained from it, possibly because they automatically activated avoidance attitudes or captured part of the residents’ cognitive resources, hindering processing of the learning material. Future studies should further explore the underlying mechanisms of this effect and how it can be diminished.

**Electronic supplementary material:**

The online version of this article (10.1007/s40037-019-00525-8) contains supplementary material, which is available to authorized users.

## What this paper adds

Postgraduate education provides residents with the opportunity to expand and refine their clinical knowledge. Residents are frequently confronted with situations that invoke negative emotions. Research has shown such emotions to potentially hinder learning, but there is no experimental evidence that this applies to residents, despite their years of training. This experiment showed that negative emotions triggered by a troublesome situation adversely influenced learning processes (study time) and outcomes (test scores) when residents studied a scientific text. Teachers should be aware of the potential adverse effect of emotions. Further research should investigate ways to protect trainees from this deleterious influence and help them take every learning opportunity to the fullest.

## Introduction

Situations that have the potential to trigger a wide range of emotions are inherent to medical practice [1]. In their daily routine, doctors and students often experience emotions such as fear of failure, sadness at a patient’s suffering, pride after a successful procedure or anger at a colleague’s unprofessional behaviour [2]. It is reasonable to expect that residents, i.e. physicians in training to become specialists, would be confronted with these emotion-evoking situations as well. They may even have to bear additional burdens, as residents often experience intimidating, undermining or humiliating behaviours, perpetrated most frequently by senior staff [3, 4], as a source of negative emotions. Such emotions are known to have deleterious consequences for residents’ wellbeing, [5] but there are reasons to suspect that emotions may also negatively affect their learning [1]. However, emotions have received little attention in medical education, and whether and how they influence learning remain unclear [6].

Defining emotion is not easy, and several concepts related to emotional experiences, such as affects and mood, are encountered in the pertinent literature [1]. In line with McConnel & Eva [1], in this manuscript we use the term ‘emotion’ in a general sense to refer to affective states and experiences. Whereas the definition of emotion is debatable, there is some agreement that emotions vary along dimensions of valence (i.e. emotions are perceived as either positive or negative, good or bad, pleasant or unpleasant) and arousal (i.e. emotions are perceived as either activating or deactivating).

Psychological research suggests that emotions can interfere with cognitive processes involved in learning, potentially hindering it. Learners’ *attention* and *perception *will ‘filter’ what they process out of the information available to them. Any factor that affects attention will impact the subsequent steps of the learning process [6]. For example, if negative information in to-be-learned material attracts the attention of learners and they do not pay sufficient attention to other parts of the material, processing the new information will be hindered, and this information will not be ‘registered’. Only when learners pay sufficient attention to the new information and use their previous knowledge to make sense of it can the new information be integrated into knowledge structures already stored in memory. This integration, known as *encoding,* will directly impact future retrieval of the information when it is needed in new situations [7].

In the present study, we were concerned with the effect of negative emotions, as they are common in the literature in medical education [1]. Negative emotions are those perceived as unpleasant or bad, when the dimension of valence is taken into consideration [1]. These emotions appear to have the potential to disrupt the above-mentioned cognitive processes through several mechanisms [8]. For example, a learner’s negative predispositions towards an event may be automatically activated by exposure to a similar event, leading the learner to—albeit unconsciously—attempt to avoid it [9]. It can also be that dealing with emotions ‘depletes’ part of the cognitive resources that would be available for learning. [10]. Apparently the same, limited set of cognitive resources employed in, for instance, understanding a new text, is applied to regulate emotions and thoughts [10, 11]. If emotions capture part of these resources, fewer resources remain for processing the text content information. [10, 11].

Although different in nature, these mechanisms and others that have been discussed in the literature [8] have in common the potential to adversely affect learning. A recent study showed that even emotions that were considered mild based on the low scores they obtained on the Positive and Negative Affect Scale (PANAS) [12] hindered undergraduate psychology students’ learning of physiological concepts [2]. However, it may be questioned whether a similar finding would apply to residents who have already gained much more experience with emotional-evoking situations and have been taught, throughout medical education, the importance of keeping their emotions under control [6]. On the other hand, it may be conjectured that residents may in fact be less resistant to emotions than undergraduate students due to their usually extremely high workload with its consequent sleep deprivation and tiredness.

The present study investigated whether negative emotions affect residents’ learning processes and learning outcomes. In a priming task, residents were exposed to a situation that developed either in a neutral way or in an emotional way. Subsequently, all residents studied a medical text, and a test was administered to measure what they had learned from it. We evaluated whether engagement in learning and learning outcomes as measured by performance in the test differed between the two groups.

## Methods

### Overview

The present study was an experiment consisting of three tasks: 1) a *priming task*, in which participants were randomly allocated to be exposed either to a video clip in an emotional version (emotional group, *n* = 29) or to a video clip in a neutral version (control group, *n* = 31); 2) a *learning task*, in which all participants studied a medical text; time spent on the task and level of cognitive engagement were measured; 3) a *testing task*, in which participants’ learning was measured by a free recall test. The three tasks were performed sequentially, in a single session.

### Participants

The participants were 60 residents (*M*_age_ = 25, SD = 2 years; 21 female) in their first week of the internal medicine residency program at the University of São Paulo Medical School, Brazil.^1^ The first week of the program includes a 3-day ‘boot camp’,[13] an in-training course aimed at facilitating the transition from undergraduate training to clinical practice. The intensive boot camp combines different approaches, especially simulation and feedback, and aims at improving essential clinical skills, doctor-patient communication, patient safety and professionalism. The activities are carried out in a friendly, safe environment and usually trigger much interest, which may explain why 60 out of the 67 recently admitted residents enrolled for the boot camp. Those who enrolled were invited by two of the authors to voluntarily participate in the study, which took place during a break in the boot camp activities. All residents accepted the invitation and were registered as participants by a person not related to the study by using an identification code that ensured anonymity.

Participants were informed that they could opt to not participate or withdraw from the study at any moment including after the data collection was completed with no consequences for their training, including assessments. A coding scheme has been used to ensure that responses were collected and analyzed anonymously. As the nature of the study prevented prior disclosure of its objectives, the residents were informed about their tasks before starting the study. After the data collection, the two groups were debriefed about the actual objective of the study, its research questions, and the importance of the study topic. All participants signed informed consent for participation in the study and for use of their data. This work was carried out in accordance with the Declaration of Helsinki: no potential harm to participants, anonymity was guaranteed, and informed consent was obtained. The study protocol was approved by the Research Ethics Committee of the São Paulo University (659.347 on 21 May 2014).

### Materials and procedure

The study was presented to the participants as two independent studies with different purposes and materials delivered in different envelopes, with a logo from two different institutions (Department of Internal Medicine and Propedeutics, Faculty of Medicine, University of Sao Paulo; Department of Internal Medicine, Academic Hospital, University of São Paulo). The rationale for this procedure was that it is known that awareness of one’s own emotions can neutralize the effect of those emotions on cognition [14], which could lead our participants to try to control that effect [15].

Before the study started, participants were randomly assigned to one of the two conditions (emotional vs. control, defined by the type of video clip used as priming task). Randomization was performed electronically by using the RANDOM Program (www.random.com/NA MEDIA®/2016). With the exception of the videos, all other materials and procedures used (see below) were the same for both groups. Participants for each condition were tested in separate auditoriums of the university building. They were requested to sit in individual chairs, leaving at least two chairs between participants, and completed the tasks individually with no communication but at the same time. Researchers and research assistants were present in the auditoriums during the whole session which lasted 60 min.

#### Priming task

The priming task consisted of a 3-minute video clip showing a resident being interviewed by a committee of senior clinicians. The committee was investigating his performance during emergency care provided to a patient who had suffered cardiac arrest and subsequently died. The clinicians conducted the interview either: i) in a cooperative and polite manner (the control version), or ii) in an accusatory and aggressive way (the emotional version). The video clip was produced to be as close as possible to a real situation: the actors were physicians dressed ‘as usual’ for their work; the medical files used were presented in the same stationery paper covers used at the hospital where they were working; the language used was ensured to be suitable for the situation by having internists who work in the department checking and amending the script for the video clip; and the office was an actual meeting room at the hospital.

In a pilot conducted prior to the study, the two versions of the video clip were tested with 8 residents from another department (4 residents saw each version). They discussed their impressions of the situation portrayed in each video in two focus groups. Both groups considered the situation to be common and realistically portrayed, but whereas the residents who watched the neutral version considered the situation sad but ‘normal’ in a resident’s life, the residents who watched the emotional version expressed negative emotions, with responses showing indignation, anxiety about how they would react and fear. The video clips were therefore considered appropriate stimuli for the study.

For ethical reasons, we used this vicarious experience to induce negative emotions in the participants instead of submitting participants to situations that could trigger real, personal emotions. Emotion-eliciting films are commonly used to evoke emotional responses in experimental settings because they offer some advantages, such as a combination of visual and auditory stimuli and standardization [15, 16] over other methods such as listening to music or the Velten approach for mood induction (briefly, reading statements and trying to feel the emotions they described) and autobiographical recall procedures [8].

Participants were asked to watch the video clip to the end and then answer six questions (see Appendix A of the online Electronic Supplementary Material). The aim of these questions was to identify whether they had previous experience with a situation similar to the one portrayed (question #1), whether they considered the situation displayed in the video realistic (question #2) and to what extent our priming task had been effective to trigger negative emotions (questions #3–6). Additional questions were asked about: age, gender, years since graduation, and number of years working before this residency.

#### Learning task

After having completed the questionnaire and handing in the first envelope (purportedly, Study 1), the participants were asked to open the second envelope (purportedly, Study *2*). This contained a medical text (930 words) on *‘Oxidative damage during cardiac arrest and cardiopulmonary resuscitation’.* The subject was chosen considering the previous knowledge participants had at this point of training (in order to avoid ceiling effects) and the connection with the video clip (priming task) since it described a patient who suffered a heart attack and did not receive treatment in time. This text was prepared by two board-certified internists^2^ who have extensive clinical and teaching experience in this residency program and are, therefore, very familiar with the residents’ level of instruction. To ensure that the text was sufficiently complex for the participants, it was also assessed by four first-year residents who were not participating in the study but were considered close to the participants in terms of knowledge base.

The instructions accompanying the medical text requested the participants to read it in the same way they would read any scientific article or chapter in a medical textbook. Participants were informed that the objective was not to read quickly, and that they had as much time as they required to complete all the tasks. A digital clock was visible in the room, and participants were asked to write down the start and end time of their reading. After studying the text, the residents were asked to answer two questions regarding their previous knowledge, if any, of the material presented in the text and whether the information was new for them.

After reading the medical text, residents were requested to move to the second page and fill in the Situational Cognitive Engagement Scale (SCES), an instrument devised and validated by Rotgans and Schmidt which is reliable for assessing involvement with learning tasks (Hancock’s coefficient H = 0.93 for exploration sample; H = 0.78 for cross-validation sample) [17]. It consists of three elements, measured by four items: 1) engagement with the task on hand (item: ‘I was engaged with the task on hand’); 2) effort and persistence (items: ‘I put in a lot of effort’; ‘I wish we could still continue with the work for a while’); and 3) having been totally absorbed by the activity (item: ‘I was so involved that I forgot everything around me’). Item responses were based on a 5-point Likert scale: 1 (Not true at all for me), 2 (Not true for me), 3 (Neutral), 4 (True for me) and 5 (Very true for me).

#### Testing task

After they had finished reading the text, participants put the text and the completed SCES back in the envelope. They were then asked to write down everything that they could remember from the text. As learning about a particular theme progresses, this particular network tends to become richer, better structured and more coherent, with more links and better integration between the new, ‘incoming’ information and the previously stored concepts. The more effectively the student has learned, the more concepts he or she will be able to recall, and recall more easily [17–19].

### Data analysis

Chi-square tests and independent t‑tests were performed for categorical and interval variables, respectively, to check whether the groups were comparable in gender, age, familiarity with situation portrayed in the video clips, perception of realism of the video and to what extent our ‘emotional manipulation’ worked on triggering emotions. Familiarity with the situation portrayed in the video was measured by the percentage of participants who responded positively to question # 1 (in the questions answered after the priming task). Perception of realism was measured by the score in question # 2. The mean score in questions # 3 to 6 (with question # 5 and 6 reversed) measured the extent to which watching the video clip triggered negative emotions, with a higher score indicating a higher level of emotions.

Independent t‑tests were performed to check for differences between the groups in two measurements of learning process: time spent in studying the text and score in the SCES.

Finally, the *learning outcomes,* as measured by the scores obtained in the free recall task, were analyzed. To establish a score for this test, a list of all relevant concepts present in the learning material (84 items) was prepared beforehand (the ‘answer key’) and agreed upon by two researchers through a consensus model. The total number of right concepts recalled (points) was the individual participant’s final score. Two researchers blindly scored 30% of the protocols. The reliability showed to be high (ICC = 0.806); then one researcher blindly scored the rest of the protocols alone. Group mean scores were computed and compared for the two experimental conditions using an independent t‑test.

For all comparisons, the level of significance was set at *p* < 0.05, and Cohen’s *d* was computed as a measure of effect size. Statistical analyses were performed with SPSS version 23 for Windows.

## Results

The age of the residents ranged from 22–34 years. There were no significant differences between participants assigned to the emotional and the control condition during the priming task: mean 25.8, 95% CI 25.01–26.58 vs mean 25.7, 95% CI 24.94–26.47, *t*(58) = 0.16, *p* = 0.88, respectively. The chi-square test showed no significant difference between the experimental conditions for gender, *χ*^2^_(1,__*N*_ _=__60)_ = 0.21, *p* = 0.64 (Tab. 1).

**Table 1:** Distribution of participants’ gender and age according to the experimental condition

		Emotional condition		Control condition	
		*n*	%	*n*	%
Gender	Male, *n* (%)	18	62	21	68
	Female, *n* (%)	11	38	10	32
Age, mean (CI)		25.8 (25.01–26.58)		25.7 (24.94–26.47)	

_*n*_ _number of students that evaluated this version;__*CI*_ _confidence interval_

Participants in the two conditions had a similar familiarity with the situation portrayed in the video clips, *χ*^2^_(1,__*N*_ _=__60)_ = 0.918, *p* = 0.338; and a similar perception of how realistic the situation was, *t*(58) = 0.14, *p* = 0.89.

The scores of emotions triggered by the two different versions of the video clip were significantly higher among participants who watched the emotion-triggering video (emotional group) than those in the control group: mean 4.5, 95% CI 4.40–4.69 vs mean 4.2, 95% CI 4.1–4.4, *t*(58) = 5.35, *p* < 0.001, respectively, demonstrating that our manipulation was effective in triggering a higher level of emotions in the emotional group relative to the neutral one.

Regarding the measures of *learning process*, participants from the two conditions differed significantly in the *time* spent studying the medical text, *t*(58) = 4.15, *p* < 0.001, *d* = 1.09 which is considered a large effect [20]. Participants in the experimental condition spent significantly less time than those in the control condition (mean 8.48, 95% CI 6.89–10.07; mean 13.94, 95% CI 11.85–16.02, respectively). However, the two conditions did not differ in their ratings of *Situational Cognitive Engagement* with the task of learning new medical material, *t(58)* = 0.72*, p* *=* 0.47 (mean 3.46, 95% CI 3.24–3.68; mean 3.57, 95% CI 3.33–3.82, respectively), (Tab. 2).

**Table 2:** Learning process and learning outcomes according to the experimental condition

	Emotional version	Control version	Statistics
	Mean (95% CI)	Mean (95% CI)	
Time spent reading material (min)	8.48 (6.89–10.07)	13.94 (11.85–16.02)	*t*(58) = 4.15, *p* < 0.001
Situational Cognitive Engagement (1–5 scale)	3.46 (3.24–3.68)	3.57 (3.33–3.82)	*t*(58) = 0.72, *p* = 0.47
Free Recall Task score	11.55 (9.14–13.96)	20.23 (16.61–23.86)	*t*(57) = 4.06, *p* < 0.001

Finally, participants’ *learning outcomes,* as measured by the scores obtained in the free recall test, showed a significant difference between the two conditions, with residents in the emotional condition (mean, 11.55, 95% CI 9.14–13.96) performing worse than their peers in the control condition (mean, 20.23, 95% CI 16.61–23.86), *t(*57) = 4.06, *p* < 0.001, *d* = 1.05 or a large effect (Tab. 2; [20]).

## Discussion

This experimental study aimed to determine whether negative emotional states influence learning processes and outcomes when residents study a scientific text. This was investigated using a video clip that was watched before the learning activity to induce either a negative or a neutral emotional state. This priming strategy proved to be effective, with residents in the emotional condition reporting more negative emotional states than those in the neutral group. Participants in the negative emotional state dedicated less time to the learning task and learned significantly less than those in the control condition.

Our findings are in line with a recent study showing that undergraduate psychology students’ learning of basic science concepts was hindered by emotional reactions [2] and suggest that residents are susceptible as well. Despite their years of undergraduate education, throughout which they certainly gained experience with dealing with emotion-invoking situations, residents’ learning also suffered an adverse effect from negative emotions. The effect was large and emerged even though the doctors were not personally involved in the seemingly threatening experience displayed in the video. This illustrates the strength of the effect and justifies concerns about its potentially deleterious consequences. Postgraduate education provides residents with the opportunity to develop their clinical skills, and expand and refine their clinical knowledge. For example, doubts arising while dealing with a particular case often lead them to search and study the scientific literature. If what residents learn decreases due to the influence of negative emotions, as the present study’s findings suggest, trainees would be missing opportunities to enrich their clinical knowledge. The final consequence of these missed opportunities would be physicians who leave their specialty training less knowledgeable, and consequently less apt to provide appropriate care to patients than they could have been. This is worrisome because trainees seem to be frequently confronted in their daily activities with situations that invoke negative emotions [6]. Our findings show that teachers should be aware of their potential adverse effects and try to minimize their occurrence. It is clear, however, that emotionally charged experiences are unavoidable in medical education, and whether there is something that can be done to help ‘protect’ trainees from their deleterious influence still requires further investigation.

Developing such protective means possibly requires a better understanding of the mechanisms through which negative emotions affect learning. These mechanisms were not the focus of the present study, but our measurements of the learning process, though far from conclusive, bring some insight into them. Under the influence of negative emotions, participants from the experimental group spent less time studying the scientific text than their colleagues from the control group, which certainly contributed to their poorer learning. The reduced time invested by the ‘emotional’ group could be explained by an ‘*avoidance response*’ triggered by watching the video. In this case, the negative emotions aroused may have acted as a sort of aversive stimulus [9], and participants would then try to ‘get away’ from the situation. However, such an avoidance response would make it reasonable to expect that participants would report less engagement with the task. This did not happen as both groups reported similar scores of cognitive engagement. The finding that both groups were equally engaged with the task speaks more in favour of the ‘resources depletion’ explanation. In fact, the same amount of perceived engagement might result in lower performance if one assumes that there is a limited ‘stock’ of cognitive resources that are employed to deal with the emotions and to study the text and make sense of the new information it presents [11]. Participants would be dedicating the same amount of cognitive efforts as those in the control group, but part of these resources will be ‘stolen’ by the demand for processing negative emotions. Our findings regarding the learning processes therefore apparently point to different explanations, and further research is needed to clarify the mechanisms through which the negative emotions hindered the residents’ learning. This could then open the door for developing and testing approaches to ‘protect’ trainees from the deleterious influence of their negative emotions.

This study has some limitations that should be addressed. First, in the present study, the emotional triggering stimulus was introduced as a separate investigation to avoid participants suspecting that we were studying their emotions, which could affect their behaviour [14]; however, it cannot be excluded that some may have considered this possibility. Second, both groups were equally familiar with the situation portrayed in the video clip and rated both versions as equally realistic in light of the situations they experience during training, but other, undetected, differences in previous experiences may have influenced the results. Because the participants were carefully randomized to the experimental conditions, this should have reduced the risk. Third, the study used a self-reported measure of emotional state, and it is unknown how well medical trainees are aware of their emotional states and are willing to honestly report them. Fourth, the control group watched a video clip portraying a situation that rather than neutral can be also emotionally charged, although less than the one used for the experimental condition. Nevertheless, if this is the case, it highlights the strength of the effect, as the difference between the two conditions would tend to increase if a completely neutral situation had been used as control. Fifth, we used a convenience sample of graduates from one school and there is no guarantee that our findings can be generalized to other contexts. Finally, we did not aim to study any particular emotion nor whether different emotions have different effects on learning or recalling. Therefore, we did not distinguish between types of emotions triggered by the manipulation, between the quality of the information learned or the duration of the effect. These remain questions to be addressed by future research.

## Conclusion

In conclusion, this study provides experimental evidence of a substantial adverse effect of negative emotions, such as those elicited by common situations during training, on the learning of residents. In addition, it suggests that the decreased learning is associated with less time dedicated to studying the material on hand. Even if the doctors reported being cognitively engaged with the learning task, they invested less time in studying and their learning was impaired compared with a control group. More research on potential explanations for the adverse effect of negative emotions on learning and on approaches to counteract this effect is required.

## Caption Electronic Supplementary Material

Manipulation Assessment Questionnaire

## Footnotes

1 In Brazil, residents have completed a 6-year undergraduate medical program and are, therefore, physicians in training to obtain a specialty degree.

2 In Brazil, medical professional associations for each medical specialty are responsible for certifying physicians as specialists through a process that involves residency training and national exams.
