# Supplementary material for: Influence of negative emotions on residents’ learning of scientific information: an experimental study
Source: Perspect Med Educ. 2019 Jul 23;8(4):209–15. doi: 10.1007/s40037-019-00525-8 (PMC6684560; doi:10.1007/s40037-019-00525-8)
Supplement: Supplementary file 1 — Manipulation Assessment Questionnaire [file 40037_2019_525_MOESM1_ESM.docx]

**APPENDIX A**

**With reference to the video clip, please answer the following questions. For the questions with a numbered scale: please mark the score that best represents your impression of the situation as experienced by the resident portrayed in the video clip.**

1. You (or someone you know) has experienced a similar situation during your medical training? Please note: it does not have to be exactly the same situation, but an experience in which the resident sees himself or herself in a situation similar to that portrayed in the video.

Yes No I am not sure

1. The video shows a realistic situation that commonly occurs during medical studies.

| 1 | 2 | 3 | 4 | 5 |
| --- | --- | --- | --- | --- |
| Totally disagree | Disagree | Neither agree nor disagree | Agree | Totally agree |

1. Do you think that watching this video helped you to understand the experience of the resident in the situation portrayed?

| 1 | 2 | 3 | 4 | 5 |
| --- | --- | --- | --- | --- |
| Not at all | To a small extent | Neither agree nor disagree | Yes, partially | Yes, very much |

1. Try to put yourself in the shoes of the first-year resident presented in this video and think about how he felt while listening to the comments of the assistant. Do you think the experience might have triggered strong emotional reactions in the resident?

| 1 | 2 | 3 | 4 | 5 |
| --- | --- | --- | --- | --- |
| Totally disagree | Disagree | Neither agree nor disagree | Agree | Totally agree |

1. Think about resident’s feelings during the experience reported in this video: the resident might have gone through this experience with feelings that can be described as:

| 1 | 2 | 3 | 4 | 5 |
| --- | --- | --- | --- | --- |
| Deeply unpleasant | Unpleasant | Neutral | Pleasant | Very pleasant |

1. How do you feel right now?

| 1 | 2 | 3 | 4 | 5 |
| --- | --- | --- | --- | --- |
| Very bad | Bad | Not good, not bad | Good | Very good |

**Please answer the following questions about yourself:**

**Age ______ Gender ________**

**Year of start of graduation _______ Year of end of graduation _______**

**Years working before start of residence ______**
